# Social Isolation Stress Induces Anxious-Depressive-Like Behavior and Alterations of Neuroplasticity-Related Genes in Adult Male Mice

**DOI:** 10.1155/2016/6212983

**Published:** 2016-01-06

**Authors:** Alessandro Ieraci, Alessandra Mallei, Maurizio Popoli

**Affiliations:** Laboratorio di Neuropsicofarmacologia e Neurogenomica Funzionale, Dipartimento di Scienze Farmacologiche e Biomolecolari e Centro di Eccellenza sulle Malattie Neurodegenerative, Università di Milano, Milano, Italy

## Abstract

Stress is a major risk factor in the onset of several neuropsychiatric disorders including anxiety and depression. Although several studies have shown that social isolation stress during postweaning period induces behavioral and brain molecular changes, the effects of social isolation on behavior during adulthood have been less characterized. Aim of this work was to investigate the relationship between the behavioral alterations and brain molecular changes induced by chronic social isolation stress in adult male mice. Plasma corticosterone levels and adrenal glands weight were also analyzed. Socially isolated (SI) mice showed higher locomotor activity, spent less time in the open field center, and displayed higher immobility time in the tail suspension test compared to group-housed (GH) mice. SI mice exhibited reduced plasma corticosterone levels and reduced difference between right and left adrenal glands. SI showed lower mRNA levels of the BDNF-7 splice variant, c-Fos, Arc, and Egr-1 in both hippocampus and prefrontal cortex compared to GH mice. Finally, SI mice exhibited selectively reduced mGluR1 and mGluR2 levels in the prefrontal cortex. Altogether, these results suggest that anxious- and depressive-like behavior induced by social isolation stress correlates with reduction of several neuroplasticity-related genes in the hippocampus and prefrontal cortex of adult male mice.

## 1. Introduction

Chronic stress is recognized to be a major risk factor for several psychiatric disorders, including anxiety and depression [1–3]. In the presence of an environmental stressor, the body responds with major modifications of the normal homeostasis, including alterations of the hypothalamic-pituitary-adrenal (HPA) axis, the major neuroendocrine system activated by stressful experiences [4, 5]. Activation of the HPA induced by stress culminates with the release of corticosterone (CORT) from the adrenal glands. These processes are finely controlled by neuronal activity of the hippocampus (HPC) and prefrontal cortex (PFC), which exert a negative feedback on the HPA axis activation. Interestingly, it has been reported that both hyper- and hypoactivation of the HPA axis are associated with increased susceptibility to developing psychiatric disorders [6, 7].

In order to better understand the etiology of depression and anxiety and to develop new treatments, valid and reliable animal models are needed [8]. Several chronic stress methods, either physical or psychosocial, have been described to promote modifications in behavior, brain structure, and neuroendocrine system in rodents [8–12]. Because psychiatric disorders in humans have been linked prevalently with social stress and/or reduced social interaction rather than physical stress [13–16], there is an increasing interest in developing chronic social stress paradigms for modeling psychiatric disorders in animal models. Different models of chronic social stress are able to induce various modifications in the behavior, brain function, and neuroendocrine system [17–20]. While it has been extensively reported that social isolation (SI) rearing induces long-lasting effects on behavior and brain structure in rodents, the behavioral consequences of SI in adult animals, especially in mice, have been less investigated [21–27]. Moreover, the molecular mechanisms underlying these modifications are still not fully understood.

Chronic stress-induced behavioral modifications have been correlated with changes of neuroplasticity-related genes in different brain areas, including neurotrophic factors, metabotropic glutamate receptors, and immediate early genes [11, 28–31]. In particular, Brain-Derived Neurotrophic Factor (BDNF), a brain plasticity marker, has been extensively investigated and associated with stress response, depression, and anxiety [31–36]. The BDNF gene is a complex gene, formed by at least eight 5′ noncoding exons, each of which can be alternatively spliced to a common 3′ protein-coding exon, to generate different transcripts [37, 38]. The complex structure of the BDNF gene allows the distinct BDNF splice variants to be differentially expressed in subcellular localizations, in specific brain regions and in response to distinct stimuli [35, 36, 38–41]. However, it is not known whether and how the BDNF splice variants are differentially regulated in the HPC and PFC of socially isolated mice.

In this study, we assessed the effect of SI stress on anxious- and depressive-like behaviors in adult male mice. Moreover, we measured the plasma CORT levels and the adrenal gland weight in socially isolated and grouped mice. Finally, we investigated whether the expression of total BDNF, BDNF splice variants, and other neuroplasticity-related genes (mGluR1, mGluR2, mGluR5, c-Fos, Arc, and Egr-1) were altered in the HPC and PFC of socially isolated compared to grouped adult male mice.

## 2. Materials and Methods

### 2.1. Animals

Male C57BL/6J mice that are 8 weeks old were purchased from Charles River (Calco, Italy) and allowed to acclimatize 1 week before being randomly divided in socially isolated (SI) and group-housed (GH) groups. Mice were maintained in a standard 12 h light/dark cycle, temperature controlled room (21 ± 1°C), with access to food and water* ad libitum*.

All animal procedures were conducted according to current regulations for animal experimentation in Italy and the European Union and were approved by the Italian Ministry of Health. To reduce the number of animals used, in agreement with the 3R guidelines, we performed behavioral and molecular analysis in the same cohort of mice. All experimental procedures involving animals were performed in accordance with the European Community Council Directive 86/609/EEC and were approved by Italian legislation on animal experimentation (Decreto Legislativo 116/1992).

### 2.2. Experimental Procedure

**Figure 1:**
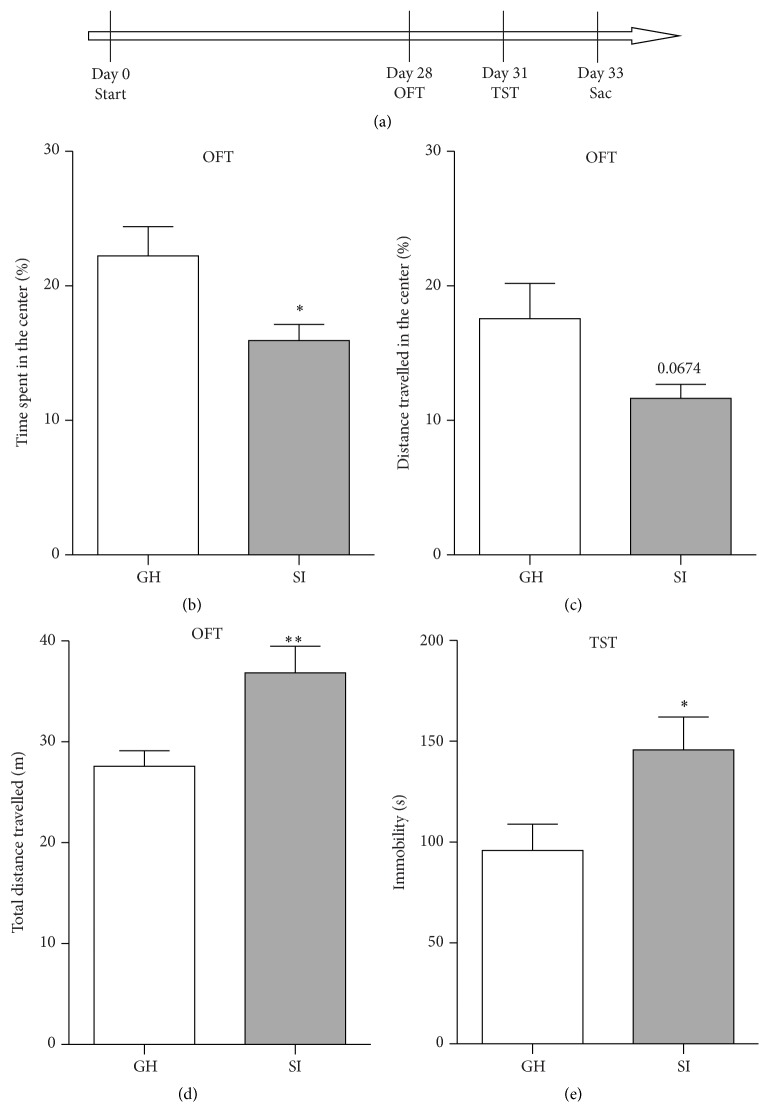
(a) Experimental schedule. Nine-week-old male mice were housed in groups (GH) or socially isolated (SI) throughout the experimental period. The open field test (OFT) and the tail suspension test (TST) were conducted on days 28 and 31, respectively. Animals were sacrificed on day 33. (b–e) Chronic adult social isolation stress induced hyperactivity and anxiety- and depressive-like behaviors in male mice. In the OFT, SI mice showed reduced percentage of time spent in the center (b), increased total distance travelled (d), and only a tendency to decreased percentage of distance travelled in the center (c). In the TST, SI mice showed increased total immobility time (e). Data are presented as mean ± SEM (*n* = 7-8 per group). ^*∗*^ *p* < 0.05; ^*∗∗*^ *p* < 0.01.

Nine-week-old male mice were individually housed (socially isolated: SI) or group-housed (4 mice per cage; group-housed: GH) in standard mouse cages. Mice were tested in the open field test (OFT) on day 28 and in the tail suspension test (TST) on day 31 (Figure 1(a)). Mice were sacrificed on day 33 and blood, adrenal glands, HPC, and PFC were harvested (Figure 1(a)).

### 2.3. Open Field Test

OFT was conducted on day 28 as described in Ieraci and Herrera [42] with some modifications. Briefly, mice were placed in the center of a 40 × 40 cm square arena divided into central (20 cm × 20 cm) and peripheral areas, with 35 cm high walls, for 10 min. The test was conducted in a dimly lit room. The total distance traveled and the time spent in the central and peripheral areas were analyzed by a video-tracking system (Any-Maze purchased by Ugo Basile, Varese, Italy).

### 2.4. Tail Suspension Test

TST was performed on day 31 as described by Steru et al. [43]. Briefly, mice were individually suspended by an adhesive tape, placed about 1 cm from the tip of the tail, on a horizontal bar 50 cm above from the bench top within a visually isolated area. A 6 min test session was videotaped and an experimenter, blinded to the experimental groups, recorded the total immobility time (defined as passive hanging without any movements except respiration).

### 2.5. Adrenal Weight

To assess whether chronic social isolation stress affects adrenal glands weight, SI and GH mice were sacrificed on day 33 and adrenal glands were removed and pruned from fat and weighed. The left and right adrenals were weighted separately for each animal. Values are reported as the adrenal/total body weight ratio (mg/g).

### 2.6. Corticosterone Assay

For CORT levels measure, trunk blood was collected on ice-cooled microcentrifuge tube containing EDTA 0.5 M pH 8.00, and plasma was separated by centrifugation and stored at −80°C. CORT levels were measured using the corticosterone ELISA Kit (Enzo Life Sciences, Florence, Italy) according to the manufacturer's instructions.

### 2.7. RNA Isolation and Reverse Transcription

Total RNA from HPC and PFC was extracted using the Direct-zol RNA MiniPrep (Zymo Research, purchased by Euroclone, Milan, Italy) according to manufacturer's instructions and then quantified by absorption at *A*_260 nm_ measured by UV spectrophotometry (NanoVue, GE Healthcare Europe GmbH, Milan, Italy). cDNA was synthesized from 1 *μ*g of DNase-treated total RNA using the iScript kit (Biorad, Milan, Italy) according to manufacturer's instructions.

### 2.8. Quantitative Real-Time PCR

qPCR analysis of mRNA expression levels was performed on a 7900HT Fast PCR System (Applied Biosystems, Monza, Italy) and iTaq Universal SYBR Green supermix (Biorad), as previously described [35]. Primers used are listed in Table 1 and were either* de novo* designed or previously published elsewhere [35, 44]. PCR cycling conditions were 10 min at 95°C, 40 cycles of 15 s at 95°C, and 1 min at 60°C. Relative expression of mRNA for the target genes was performed by the comparative C_T_  (ΔΔC_T_) method using *β*-actin and GAPDH as control reference genes. The relative mRNA levels were expressed as fold change. Analysis of melting curve verified the specificity of the PCR products.

### 2.9. Western Blot

Western blot was performed as previously described [45]; briefly HPC and PFC were lysed in ice-cold RIPA buffer (0.15 mM NaCl, 0.05 mM Tris_HCl, pH 7.2, 1% Triton X-100, 1% sodium deoxycholate, and 0.1% SDS) with Protease Inhibitor Cocktail (Sigma-Aldrich, Milan, Italy). Protein concentration was determined by the Quantum Bicinchoninic Protein Assay (Euroclone) and 15 *μ*g of proteins was separated on SDS-PAGE gels and transferred to a polyvinylidene difluoride membrane (GE Healthcare, Amersham, Milan, Italy), followed by blocking in 5% milk dissolved in Tris Buffer Saline-Tween 20 (TBST). Membranes were incubated with anti-BDNF (1 : 2,000, rabbit polyclonal, Alomone, Jerusalem, Israel) or anti-*β*-actin (1 : 20,000, mouse monoclonal, Sigma) antibodies. After washing with TBST, filters were incubated with peroxidase-conjugated secondary anti-rabbit (1 : 3,000, Sigma-Aldrich) or with the fluorescent IRDye secondary anti-mouse antibody (LI-COR, purchased from Carlo Erba Reagents, Milan, Italy). Peroxidase immunoreactivity bands were revealed by chemiluminescence using ECL detection system (Biorad). Chemiluminescence and fluorescence membrane signals were scanned and quantified in an Odyssey LI-COR scanner (Carlo Erba Reagents).

### 2.10. Statistical Analysis

Statistical analyses were performed using GraphPad Prism 6 (GraphPad Software, La Jolla, CA). Data are presented as mean ± standard error of the mean (SEM). Statistical analyses were made using unpaired *t*-test or two-way analysis of variance (ANOVA), and LSD procedure was used for multiple comparison analysis. Pearson's *r* correlations were performed to assess the correlation between CORT levels, gene expression levels, and behavioral analyses (Tables 2–7).

## 3. Results

### 3.1. Social Isolation Stress Induces Behavioral Impairments in Adult Male Mice

To assess whether social isolation induces modifications of emotional behavior in adult mice, 9-week-old male mice were randomly housed in group (GH) or socially isolated (SI) for 4 weeks and then tested in the OFT and in the TST to measure the anxious- and depressive-like behaviors, respectively (Figure 1(a)). SI mice spent significantly less time in the center of the arena (*p* = 0.029) (Figure 1(b)) and showed a tendency to a reduced distance travelled in the center (*p* = 0.067) (Figure 1(c)), indicating anxious-like behavior. Interestingly, SI mice showed an increased level of total distance travelled in the OFT as compared to the GH mice (*p* = 0.007) suggesting a hyperactivity phenotype in SI mice (Figure 1(d)). Moreover, in the TST, the SI exhibited a significantly higher period of immobility time compared to the GH mice (*p* = 0.03), indicating a depressive-like behavior in SI mice (Figure 1(e)).

### 3.2. Corticosterone Plasma Levels Are Decreased in Socially Isolated Adult Male Mice

**Figure 2:**
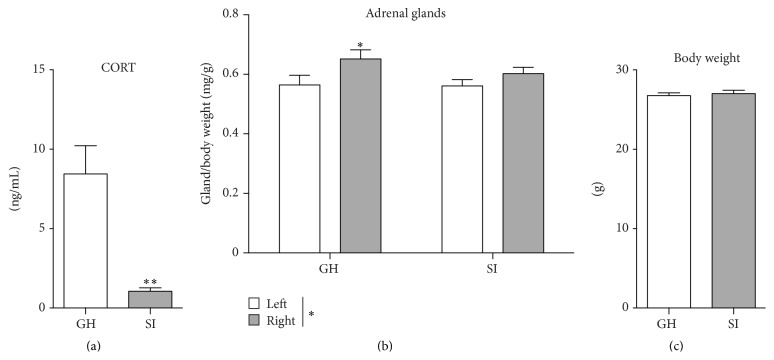
Chronic social isolation stress in adulthood reduces plasma corticosterone levels. (a) Corticosterone levels were reduced in the plasma of socially isolated adult male mice. (b) Socially isolated mice showed reduced differences between the left and right adrenal/body weight ratio. (c) There were no differences in the total body weight between group-housed (GH) and socially isolated (SI) mice. Data are presented mean ± SEM (*n* = 7-8 per group). ^*∗*^ *p* < 0.05; ^*∗∗*^ *p* < 0.01.

To study whether the behavioral changes induced by social isolation are associated with alterations of the HPA axis function, we measured the plasma CORT levels and the adrenal glands weight, as an index of terminal phase of the HPA activation, in the SI and GH mice. Interestingly, the levels of plasma CORT were markedly reduced in the SI mice compared to GH mice (*p* = 0.002) (Figure 2(a)). Moreover, a negative Pearson's correlation was specifically detected between the immobility time measured in the TST and the plasma CORT levels (*r* = −0.6367; *p* = 0.0107; Table 2). A two-way ANOVA showed a significant overall effect for the size of the adrenal glands (*F*_(1,26)_ = 5.704; *p* = 0.0245), with the left adrenal gland heavier than the right one (Figure 2(b)), consistent with the data reported in literature [46, 47]. A further Fisher's LSD* post hoc* analysis revealed a significant difference between the right and left adrenal gland for the GH mice (*p* = 0.03), but not for the SI mice (*p* = 0.19) (Figure 2(b)). These differences were not due to changes in body weight (*p* = 0.45) (Figure 2(c)).

### 3.3. Neuroplasticity-Related Genes Are Reduced in the HPC and PFC of Socially Isolated Adult Male Mice

**Figure 3:**
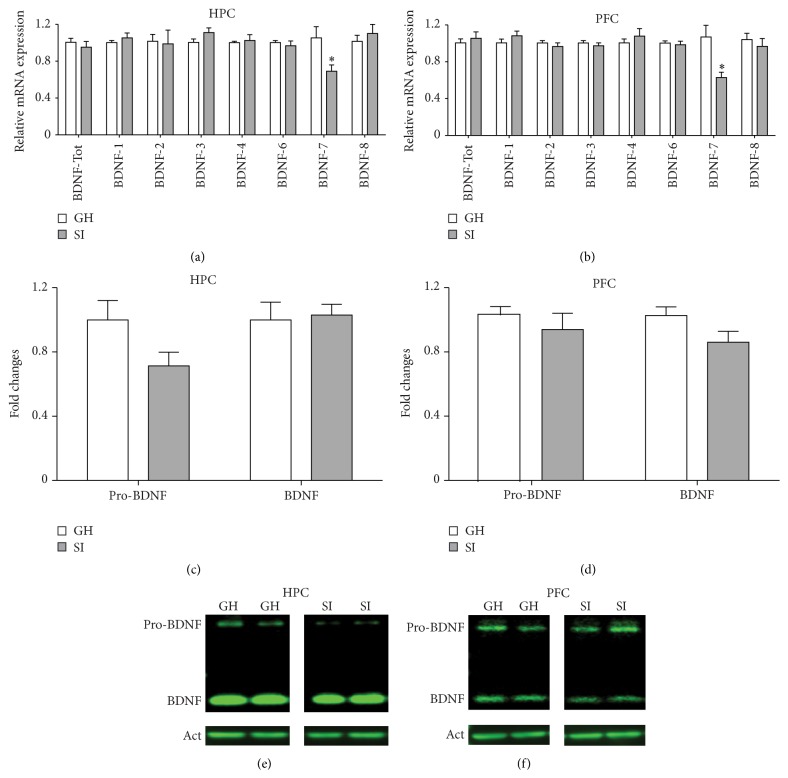
Social isolation stress in adulthood reduces BDNF-7 splice variants in the hippocampus (HPC) and prefrontal cortex (PFC). (a-b) Adult socially isolated mice showed decreased levels of BDNF-7 mRNA transcript in the HPC (a) and PFC (b). On the contrary, total BDNF and BDNF splice variants 1, 2, 3, 4, 6, and 8 were not modulated by adult SI stress. Data are presented as Mean ± SEM (*n* = 7-8 per group). ^*∗*^ *p* < 0.05. (c–f) Pro-BDNF and mature BDNF protein levels in the HPC (c, e) and prefrontal cortex (d, f). Densitometric quantifications were obtained as ratio of Pro-BDNF/*β*-actin and BDNF/*β*-actin. Data are presented as mean ± SEM (*n* = 6 per group). (e-f) Representative Western blot pictures from Pro-BDNF, BDNF, and *β*-actin.

To investigate the possible molecular mechanisms underlying the social isolation stress-induced behavioral changes, we evaluated the mRNA levels of different genes involved in neuronal plasticity in the HPC and PFC of SI and GH mice. Firstly, we measured the mRNA levels of total BDNF, which is known to modulate stress-induced behavioral changes [34], and BDNF splice variants 1, 2, 3, 4, 6, 7, and 8. We found that BDNF-7 transcript was significantly and selectively downregulated in both the HPC and the PFC of SI mice, compared to GH mice (HPC: *p* = 0.028; PFC: *p* = 0.01) (Figure 3), while no significant differences were revealed for total BDNF and all the other BDNF transcripts analyzed (Figure 3). Protein levels analysis by Western blot revealed only a trend for reduction of pro-BDNF in the HPC (*p* = 0.082; Figure 3(c)) and mature BDNF in the PFC (*p* = 0.085; Figure 3(d)).

Interestingly, we have observed a significant positive correlation between the plasma CORT levels and the BDNF-7 mRNA levels measured in the HPC, with only a trend in the PFC (HPC: *r* = 0.5552; *p* = 0.0317; PFC: *r* = 0.4933; *p* = 0.0522; Table 3). Total BDNF mRNA levels in the HPC and BDNF-7 mRNA levels in the PFC positively correlate with the percentage of time spent in the OF center (total BDNF: *r* = 0.5459; *p* = 0.0353; BDNF-7: *r* = 0.5886; *p* = 0.021; Table 4). Moreover, we found a trend for correlation between the cortical BDNF-7 levels and the total distance travelled in the OF (*r* = −0.5107; *p* = 0.0517; Table 6).

**Figure 4:**
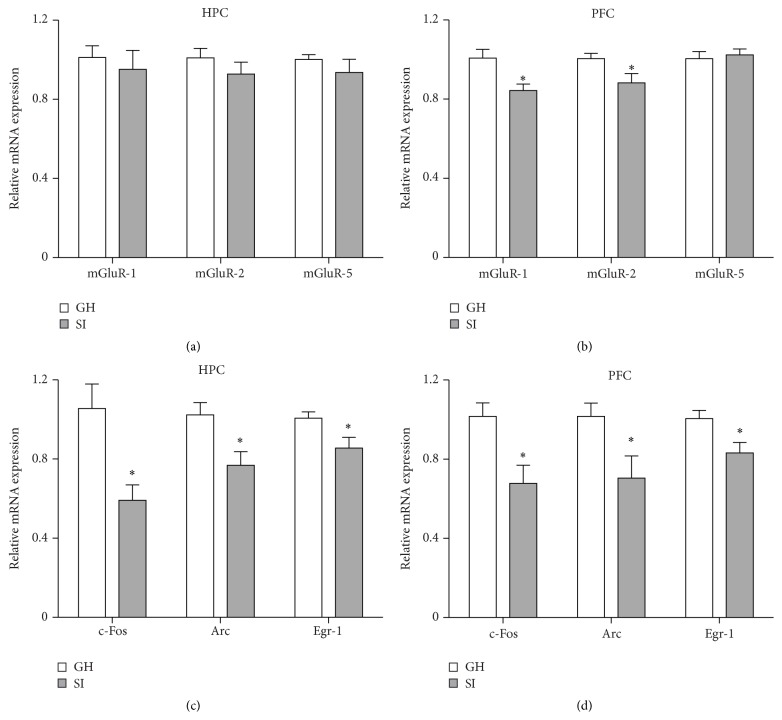
Social isolation stress in adulthood decreases expression of neuroplasticity-related gene in the hippocampus (HPC) and prefrontal cortex (PFC). (a, b) In socially isolated (SI) adult male mice, mGluR1 and mGluR2 mRNA levels were reduced in the PFC (b). No differences were revealed for mGluR5 in the PFC and for mGluR1, mGluR2, and mGluR5 in the HPC (a-b). SI mice showed reduced mRNA levels of c-Fos, Arc, and Egr-1 in the HPC and PFC (c-d). Data are presented as mean ± SEM (*n* = 7-8 per group). ^*∗*^ *p* < 0.05.

Several lines of evidence suggest that metabotropic glutamate receptors are involved in stress-related disorders and in the mechanisms of action of antidepressants [48–50]. Thus, we sought to investigate whether behavioral modifications induced by SI stress correlated with changes in the expression of mGluR1, mGluR2, and mGluR5 in the HPC and PFC. Interestingly, we found that mGluR1 and mGluR2 mRNA levels were significantly reduced in the PFC of SI mice compared to GH mice (mGluR1: *p* = 0.012; mGluR2: *p* = 0.038; Figure 4(b)), while no differences were revealed for mGluR5 mRNA levels in the PFC and for all three mRNA levels in the HPC between SI and GH mice (Figures 4(a)-4(b)). However, only mGluR1 in the PFC showed a positive correlation with the percentage of time spent in the OF center (*r* = 0.5886; *p* = 0.021; Table 4) and the distance travelled in the OF center (*r* = 0.5886; *p* = 0.021; Table 5).

Immediate early genes (IEGs) are genes rapidly expressed upon different stimuli and play important roles in synaptic plasticity [51]. However, constitutive changes in IEGs expression have been reported also after chronic stress and antidepressant treatments [52–54]. Therefore, to investigate whether SI induced alterations in the IEGs expression, we evaluated the mRNA levels of c-Fos, Arc, and Egr-1 in the HPC and PFC of SI and GH mice. We found that mRNA levels of c-Fos, Arc, and Egr-1 were decreased in both HPC and PFC of SI mice compared to GH mice (HPC: c-Fos: *p* = 0.0092; Arc: *p* = 0.016; Egr-1: *p* = 0.027; PFC: c-Fos: *p* = 0.0099; Arc: *p* = 0.029; Egr-1: *p* = 0.69) (Figures 4(c)-4(d)). A significant positive correlation was found for c-Fos and Arc mRNA levels in the HPC and PFC and the percentage of time spent in the OF center (HPC: c-Fos: *r* = 0.6627; *p* = 0.0071; Arc: *r* = 0.536; *p* = 0.0395; PFC: c-Fos: *r* = 0.5797; *p* = 0.035; Arc: *r* = 0.5826; *p* = 0.0227; Table 4).

## 4. **Discussion**

In the present study, we have found that SI adult male mice spend less time in the center and are hyperactive in the OFT. Additionally, SI mice showed higher immobility time in the tail suspension test compared to GH mice. Although several studies have reported that SI rearing induces hyperactivity and anxious- and depressive-like behavior in rodents, only a few studies have addressed the consequences of SI in adult rodents, especially in mice [21, 23, 55–59]. Our findings suggest that social deprivation may be deleterious not only during childhood and adolescent period but also during adulthood in male mice. Moreover, our results are consistent with previous data showing that stress induces hyperactivity in rodents in response to exposure to novel environments, such as an open field [52, 60–63].

The reduced plasma CORT levels measured in SI adult mice are paralleled by the decreased difference in weight between the left and right adrenal glands, suggesting that this hypofunction may be due to morphological changes in the HPA axis. Although stress is usually correlated with HPA hyperactivity, our results are in line with previous reports showing reduced levels of CORT in SI adult male rat and female mice [55, 56]. Moreover, lower adrenal activity in response to chronic stress was also reported in social defeat animal models [64]. Interestingly, reduced basal levels of CORT have been reported in patients with posttraumatic stress disorder (PTSD), even decades after the traumatic events [65–67]. Clinical studies have also reported lower CORT levels in some patients at risk for PTSD and in healthy subjects living under constantly stressful environments [68, 69]. Intriguingly, it has been reported that rats with reduced basal levels of CORT display increased anxious-like behavior and low extinctions rates of conditioned fear, behavioral disturbances that model some aspect of PTSD [70]. Delayed and incomplete contextual fear extinction has been also described in SI adult mice [23]. Altogether these lines of evidence suggest that social isolation stress in adult mice could be a suitable model to study the behavioral and molecular alterations related to PTSD [23]. However, further studies will be necessary to fully characterize this model.

The results showing that SI stress in adult male mice downregulated the expression of IEGs are consistent with previous reports showing that social isolation rearing reduced IEGs in rats [52, 71, 72]. IEGs are defined by their capability to be quickly transcribed without new protein synthesis, activated by transcription factors that are regulated by phosphorylation. We measured the levels of gene expression 2 days after the TST to limit the possibility that behavioral tests affected the gene expression. Therefore, the downregulation of IEGs observed here likely reflects an adaptation to the chronic SI stress used, rather than the behavioral manipulation effects. However, we cannot exclude that the downregulation observed is due to the sum of social isolation and behavioral test manipulations.

We found that SI stress decreased Arc expression in both of the HPC and PFC. It has been previously reported that Arc mRNA is delivered to dendrites, where it is specifically accumulated at recently activated synapses and locally translated into protein [73]. Arc has a critical role in neuroplasticity and behavioral processes [74]. Our finding that reduced Arc expression correlated with increased anxiety- and depression-like phenotypes in SI adult mice is consistent with previous data showing that Arc expression is higher in the low-anxious Sprague-Dawley rats compared to the highly anxious hooded PVG strain and that chronic antidepressant treatment increased Arc expression [54, 75]. Altogether, these data suggest that Arc may have a direct role in the synaptic plasticity responses after activation and in behavioral processes, coupling neuronal activity with structural remodeling and functional changes [76, 77]. Interestingly, Egr-1 is a transcriptional factor directly controlling the expression of Arc that has also been associated with some form of synaptic plasticity and may be required for the development of late long-term potentiation and behavioral responses [78–80]. The present results, showing that both Egr-1 and Arc are decreased in HPC and PFC, may suggest that Arc is downregulated as a consequence of stress-induced reduction of Egr-1 expression.

Remarkably, we found that BDNF-7 transcript was selectively reduced in both HPC and PFC of SI mice, while the levels of total BDNF and all other exons were not changed. As a rule, BDNF-7 mRNA levels are lower compared to the most abundant BDNF transcripts in the brain (1, 2, 4, and 6); therefore the reduction of BDNF-7 levels observed in SI mice is probably not enough to significantly reduce the total expression of BDNF (which includes all transcripts). However, the BDNF-7 transcript is more efficiently translated to protein, compared to more highly expressed BDNF transcripts 1, 4, and 6 [81] and is one of the few BDNF transcripts to be present in the dendrites in basal conditions [82]. It has been reported that synaptic plasticity controls local dendritic BDNF translation and that alterations in this pathway may compromise the long-lasting spine plasticity and lead to behavioral dysfunctions [83]. Taken together, these lines of evidence strongly suggest that BDNF-7 may be rapidly translated in the dendritic compartment upon synaptic activation and that reduction of BDNF-7 levels induced by SI stress may contribute to the behavioral impairments observed in SI adult mice.

Our results showing that SI stress decreased the levels of mGluR1 and mGluR2 in the PFC are in line with previous data reporting a significant attenuation in mGluR function in the PFC of SI reared rats [84]. Remarkably, it has been also reported that chronic unpredictable stress (CUS) reduces mGluR2 expression and that upregulation of mGluR2 reverses the anxious- and depressive-like behaviors caused by CUS [48, 50]. Furthermore, a possible role of mGluR2 in controlling hyperactivity has been described as well. Systemic mGluR2/3 agonist administration reverses the SI rearing-induced hyperactivity [85]. Moreover, injecting a mGluR2/3 agonist locally into the PFC blocks the amphetamine-induced hyperactivity [86]. Altogether, these data indicate that downregulation of mGluR2 levels in the PFC of SI mice may contribute to the behavioral impairments observed. Although previous studies have reported behavioral and synaptic plasticity deficits in the mGluR1 null mice [87, 88] and mGluR1/5 agonist reduced the anxiety-like behaviors of DBA/2 mice [89], the possible role of mGluR1 in the PFC has not been specifically investigated yet. Further studies will be necessary to assess whether the expression of mGluR1 in the PFC contributes to the behavioral changes induced by SI.

We specifically found a reduction of mGluR1 and mGluR2 in the PFC but not in the HPC. However, since we have analyzed gene expression in the entire HPC, without subdivision of the different regions (dorsal and ventral hippocampus, dentate gyrus, CA1, and CA3), it is possible that variations in specific regions may be attenuated from the expression in the other regions and therefore not detected by our analyses. Further studies will be necessary to uncover whether changes of the mGluR1 and mGluR2 expression in specific regions of the HPC are actually induced by social isolation. Nevertheless, differential modulation of gene expression in different brain areas is quite common and it has been described for several genes and environmental manipulations.

A major limitation of the present study is the descriptive nature of our analysis. Although we have found significant correlations between behavioral impairments, CORT plasma levels, and gene expression levels in the HPC and PFC, these findings are not sufficient to clearly establish a direct causal effect between these molecular changes and the behavioral alterations. Further studies using both molecular and pharmacological approaches, to specifically modulate the expression of these genes in HPC and PFC, will be necessary. Another limitation of the study is the lack on of time-dependent effects of SI. We have found behavioral and molecular changes after 4 weeks of social isolation stress. It would be interesting in future studies to investigate the shortest period of SI that is able to induce behavioral and molecular changes in adult mice and whether a longer period of isolation is able to worsen the phenotype that we have reported here. Moreover, while we cannot rule out that the molecular changes observed in SI mice are a consequence of the combination of the social isolation paradigm and the behavioral tests, our results show that neuroplasticity-related genes are differentially modulated in GH and SI mice.

Overall, in the present study we showed that SI stress in the adult male mice resulted in anxiety- and depression-like phenotypes. Additionally, we showed that behavioral modifications correlated with decrease of plasma CORT levels and downregulation of neuroplasticity-related genes in HPC and PFC. In particular, the selective reduction of the BDNF-7 transcript in both brain areas of SI mice for the first time links this dendrite-resident BDNF isoform with synaptic plasticity and the behavioral consequences of SI. Further work to explore the role of this BDNF transcript is warranted.

## Figures and Tables

**Table 1:** List and sequence of primers used in this study.

Gene	Forward	Reverse
*Real-time PCR*		
BDNF-1^a^	CCTGCATCTGTTGGGGAGAC	CGCCTTCATGCAACCGAAGTAT
BDNF-2^a^	ACCTTTTCCTCCTCCTGCG	TGGATGAAGTACTACCACCTCGG
BDNF-3^a^	TGAGACTGCGCTCCACTCCC	CGCCTTCATGCAACCGAAGTAT
BDNF-4^a^	CAGAGCAGCTGCCTTGATGTTT	CGCCTTCATGCAACCGAAGTAT
BDNF-6^a^	ACAATGTGACTCCACTGCCGG	CGCCTTCATGCAACCGAAGTAT
BDNF-7^a^	ACTTACAGGTCCAAGGTCAACG	GGACAGAGGGTCGGATACAG
BDNF-8^a^	ATGACTGTGCATCCCAGGAGAAA	CGCCTTCATGCAACCGAAGTAT
BDNF^a^	TCGTTCCTTTCGAGTTAGCC	TTGGTAAACGGCACAAAAC
mGluR1	CACAGCCCTTGCCAAAGAGAATGAG	CACTCCACTCGAGGTTAACGGA
mGluR2	CACCACCTGTATCATCTGGCTG	GAGCACCACAGAGCCACTGA
mGluR5	AGACGACCTGGCCAAACAAA	CTACTGCTCATGAAAGCCCACA
c-Fos^b^	CTGCAGCCAAGTGCCGGAATC	GGCAATCTCAGTCTGCAACGC
Arc	AGCCCAAACTCAAGCGCTTT	GAAGGCTCAGCTGCCTGCCTC
Egr-1^b^	CCTTCAATCCTCAAGGGGAGC	AACCGAGTCGTTTGGCTGGGA

*β*-Actin^a^	GCCAGAGCAGTAATCTCCTTCT	AGTGTGACGTTGACATCCGTA
GAPDH^a^	CGTGCCGCCTGGAGAAACC	TGGAAGAGTGGGAGTTGCTGTTG

^a^Ieraci et al., 2015 Hippocampus [35]; ^b^Rusconi et al., 2015 Cereb. Cortex [44].

**Table 2:** Pearson's correlation between corticosterone levels and behavioral analysis.

	HPC	
	*r*	*p*
% time spent in the center	0.1178	0.6759
% distance in the center	−0.07975	0.7776
Total distance travelled	−0.4050	0.1343
Tail suspension test	**−0.6367**	**0.0107**

**Table 3:** Pearson's correlation between corticosterone and gene expression levels.

	HPC		PFC	
	*r*	*p*	*r*	*p*
Total BDNF	0.1281	0.6490	−0.1253	0.6438
BDNF-1	0.01566	0.9558	−0.04050	0.8816
BDNF-2	0.003361	0.9905	0.2474	0.3556
BDNF-3	−0.4312	0.1085	0.2589	0.3330
BDNF-4	−0.06950	0.8056	−0.1629	0.5467
BDNF-6	0.1259	0.6549	0.1538	0.5695
BDNF-7	**0.5552**	**0.0317**	**0.4933**	**0.0522**
BDNF-8	−0.09168	0.7452	0.2692	0.3133
mGluR1	0.07396	0.7934	0.3207	0.2439
mGluR2	0.01753	0.9506	0.3320	0.2267
mGluR5	0.2400	0.3890	−0.4608	0.0839
c-Fos	0.3787	0.1639	0.4177	0.1214
Arc	0.4144	0.1246	0.3985	0.1412
Egr-1	0.3721	0.1720	0.4619	0.0830

**Table 4:** Pearson's correlation between the percentage time spent in the open field center and gene expression levels.

	HPC		PFC	
	*r*	*p*	*r*	*p*
Total BDNF	**0.5459**	**0.0353**	−0.01596	0.9550
BDNF-1	−0.06687	0.8128	−0.3120	0.2576
BDNF-2	0.3161	0.2510	0.5469	0.0349
BDNF-3	−0.1448	0.6066	0.08998	0.7498
BDNF-4	0.07657	0.7862	−0.1619	0.5643
BDNF-6	0.4217	0.1175	0.1034	0.7138
BDNF-7	0.3897	0.1510	**0.5886**	**0.0210**
BDNF-8	−0.4328	0.1071	−0.02065	0.9418
mGluR1	0.08381	0.7665	**0.6689**	**0.0064**
mGluR2	0.3911	0.1495	0.4856	0.0665
mGluR5	0.2127	0.4465	0.3362	0.2205
c-Fos	**0.6627**	**0.0071**	**0.5797**	**0.0235**
Arc	**0.5360**	**0.0395**	**0.5826**	**0.0227**
Egr-1	0.3196	0.2456	0.5121	0.0510

**Table 5:** Pearson's correlation between the percentage distance travelled in the open field center and gene expression levels.

	HPC		PFC	
	*r*	*p*	*r*	*p*
Total BDNF	0.2967	0.2829	−0.2008	0.4731
BDNF-1	−0.1696	0.5456	−0.4613	0.0835
BDNF-2	0.005426	0.9847	0.1432	0.6106
BDNF-3	−0.05189	0.8543	0.09637	0.7326
BDNF-4	0.02956	0.9167	−0.3317	0.2271
BDNF-6	0.2578	0.3536	−0.08952	0.7510
BDNF-7	0.1517	0.5895	0.3941	0.1461
BDNF-8	−0.2511	0.3668	0.01725	0.9514
mGluR1	0.1989	0.4772	**0.5179**	**0.0480**
mGluR2	0.3364	0.2202	0.4008	0.1388
mGluR5	0.2203	0.4302	0.4895	0.0640
c-Fos	0.3250	0.2373	0.3312	0.2278
Arc	0.2202	0.4303	0.2744	0.3222
Egr-1	0.1294	0.6458	0.2010	0.4727

**Table 6:** Pearson's correlation between the total distance travelled in the open field and gene expression levels.

	HPC		PFC	
	*r*	*p*	*r*	*p*
Total BDNF	−0.01723	0.9514	0.2982	0.2804
BDNF-1	0.4873	0.0654	0.3025	0.2731
BDNF-2	−0.06728	0.8117	0.1369	0.6266
BDNF-3	0.5139	0.0500	0.3978	0.1420
BDNF-4	0.3485	0.2030	0.1766	0.5289
BDNF-6	0.1649	0.5571	0.2295	0.4107
BDNF-7	−0.3760	0.1672	**−0.5107**	**0.0517**
BDNF-8	0.1553	0.5806	0.09135	0.7461
mGluR1	−0.1733	0.5367	−0.4017	0.1378
mGluR2	0.1448	0.6066	−0.3355	0.2215
mGluR5	0.03919	0.8897	−0.02266	0.9361
c-Fos	−0.4212	0.1180	−0.3366	0.2200
Arc	−0.3860	0.1553	−0.2858	0.3017
Egr-1	−0.1496	0.5947	−0.4556	0.0879

**Table 7:** Pearson's correlation between the immobility time measured in the tail suspension test and gene expression levels.

	HPC		PFC	
	*r*	*p*	*r*	*p*
Total BDNF	−0.2868	0.3000	−0.3003	0.2768
BDNF-1	0.1625	0.5628	−0.2971	0.2822
BDNF-2	0.1307	0.6425	−0.1610	0.5665
BDNF-3	**0.6580**	**0.0077**	−0.3290	0.2311
BDNF-4	0.2176	0.4359	−0.2689	0.3325
BDNF-6	−0.1178	0.6759	−0.3714	0.1729
BDNF-7	−0.1460	0.6036	−0.3611	0.1860
BDNF-8	0.1389	0.6215	0.1938	0.4888
mGluR1	0.1182	0.6748	−0.1393	0.6205
mGluR2	0.00844	0.9762	−0.05227	0.8532
mGluR5	0.1913	0.4947	0.2808	0.3107
c-Fos	−0.2720	0.3267	−0.2640	0.3417
Arc	−0.3338	0.2241	−0.2845	0.3040
Egr-1	−0.09785	0.7286	−0.1392	0.6208
